# Effects of industrial effluents on the quality of water in Namanve stream, Kampala Industrial and Business Park, Uganda

**DOI:** 10.1186/s13104-020-05061-x

**Published:** 2020-04-16

**Authors:** Christopher Angiro, Patrick P’Odyek Abila, Timothy Omara

**Affiliations:** 1 Department of Chemistry, Faculty of Science, Kyambogo University, P. O. Box 1, Kyambogo, Kampala, Uganda; 2 National Livestock Resources Research Institute, National Agricultural Research Organization (NARO), P. O. Box 5704, Nakyesasa, Kampala, Uganda; 3 Department of Chemistry and Biochemistry, School of Biological and Physical Sciences, Moi University, Uasin Gishu County, P.O. Box 3900-30100, Eldoret, Kenya; 4 Department of Quality Control and Quality Assurance, Product Development Directory, AgroWays Uganda Limited, Plot 34-60, Kyabazinga Way, P.O. Box 1924, Jinja, Uganda; 5 Africa Center of Excellence II in Phytochemicals, Textiles and Renewable Energy (ACE II PTRE), Moi University, Uasin Gishu County, P.O. Box 3900-30100, Eldoret, Kenya

**Keywords:** Biochemical oxygen demand, *Escherichia coli*, Conductivity, Total dissolved solids, Total suspended solids, Wakiso district

## Abstract

**Objective:**

Kampala Industrial and Business Park (KIBP) is one of the premier and the most successful Ugandan industrial complexes that impact the inner Murchison bay of Lake Victoria. The current study aimed at evaluating the effect of industrial effluents on the physicochemical and microbiological quality of water taken from four different sites along Namanve stream in KIBP, Wakiso district, Uganda.

**Results:**

All the water quality parameters were below WHO maximum permissible limits except turbidity, electrical conductivity and *Escherichia coli* count. Mean values of the monitored water quality parameters increased from the point of effluent discharge downstream of Namanve stream.

## Introduction

Uganda’s 2030 vision to industrialize for economic transformation, recover its economic status lost in the 1970s and attain a middle-income status has presented numerous environmental challenges [1–3]. Industries are generating volumetric wastes which are discharged without treatment into nearby water bodies, potentially degrading their water quality [2, 4–7]. Most industries in Uganda use outdated manufacturing technologies and do not have functional effluent treatment plants. Therefore, raw and harmful wastes are discharged into the surrounding water bodies [2, 5, 7].

As part of continuous assessment of environmental quality in peri urban Kampala and other industrial complexes of Uganda [4, 8–11], the current study investigated the physicochemical and microbiological profile of water from a stream serving one of the premier and the best planned industrial complexes in the history of Uganda.

## Main text

### Method

**Fig. 1:**
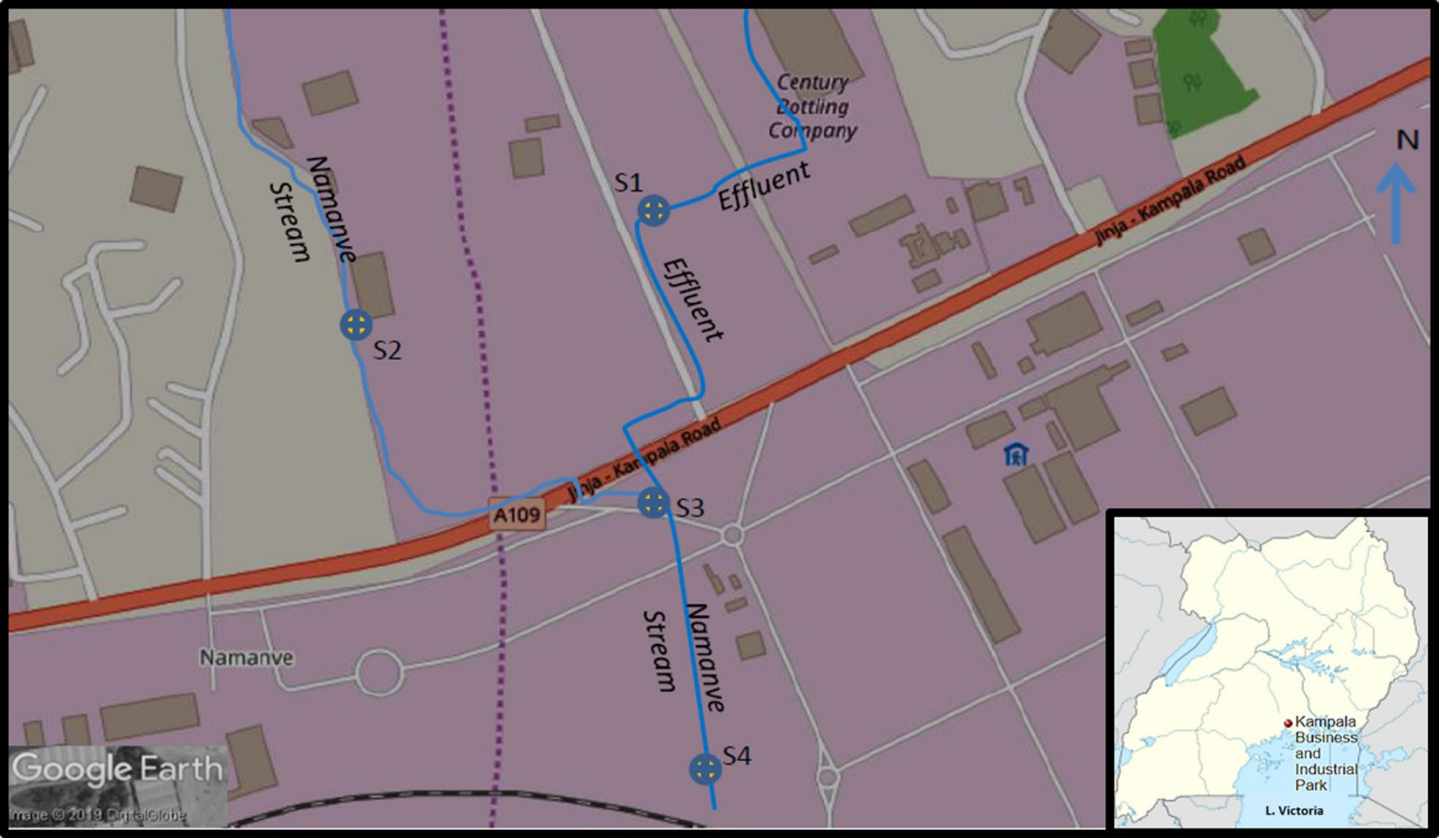
Map of KIBP showing the sampled sites: S1(Fresh water on the stream before effluent discharge), S2 (Effluent from industry), S3 (point of effluent discharge into the stream) and S4 (mixture of effluent and fresh water). Inset is the map of Uganda showing the location of KIBP in Uganda (Adapted from Wikipedia)

The study was done on water samples from Namanve stream, Kampala Industrial and Business Park (KIBP) in Wakiso district of Uganda. KIBP lies at coordinates 0° 20′ 35.0″ N, 32° 41′ 55.0″ E, about 15 km East of Kampala, the capital city of Uganda [4, 12]. Namanve stream originates from Namanve wetlands and it is among the streams that flow into the inner Murchison bay of Lake Victoria [13]. The major industries near this stream are beverage industries such as Century Bottling company and Rwenzori Bottling company (Fig. 1).

#### Materials and study approval

Apparatus and reagents were those previously used [8, 10, 14]. Study approval was granted by Department of Chemistry, Kyambogo University, Uganda (Approval No. 16/U/14048/BMD).

#### Sampling and analysis of water

Water samples were taken from four different sites (S1 to S4) between April 2019 and May 2019 (Fig. 1). The first sampling was conducted during dry weather conditions while the second sampling was conducted after days of down pours. All sampling was done between 10:00 a.m. and 11:30 a.m. (East African Standard Time) as described by Omara et al. [8].

The pH, electrical conductivity and total dissolved solids (TDS) of water samples were determined on-site [10]. Turbidity in Formazin Turbidity Unit (FTU) was measured using a double beam optimal geometry Genesys 10S UV–Vis spectrophotometer (Thermo Scientific, USA) [10, 15].

Total Suspended Solids (TSS) was determined by gravimetry. Measured 50 mL of each sample was filtered using a pre-weighed filter paper (Qualitative circles, 125 mm Ø). The residue retained on the filter paper was oven dried for 4 h to a constant weight at 105 °C. The weight of the filter paper and the residue were determined. The difference in the weights of the filter paper and the filter paper plus the residue represented the TSS.

Total nitrogen (TN), biochemical oxygen demand (BOD) and total phosphate levels were determined following APHA method [16]. Microbial analysis was done for *Escherichia coli* using APHA method [17] described before [10, 18].

#### Analytical quality assurance and quality control

All reagents used were of high analytical purity. Equipment used were calibrated prior to use. Quality control was achieved through analysis of all samples at least in duplicate.

#### Statistical analysis

Data were captured in Excel and checked for normality using Shapiro–Wilk test. Non-parametric multivariate tests were performed as the data did not follow a normal distribution. Thus, the data were presented pictorially as box plots. The analyses were performed at a 95% confidence interval using R for statistical analysis (R Core team, 2013).

### Results

**Fig. 2:**
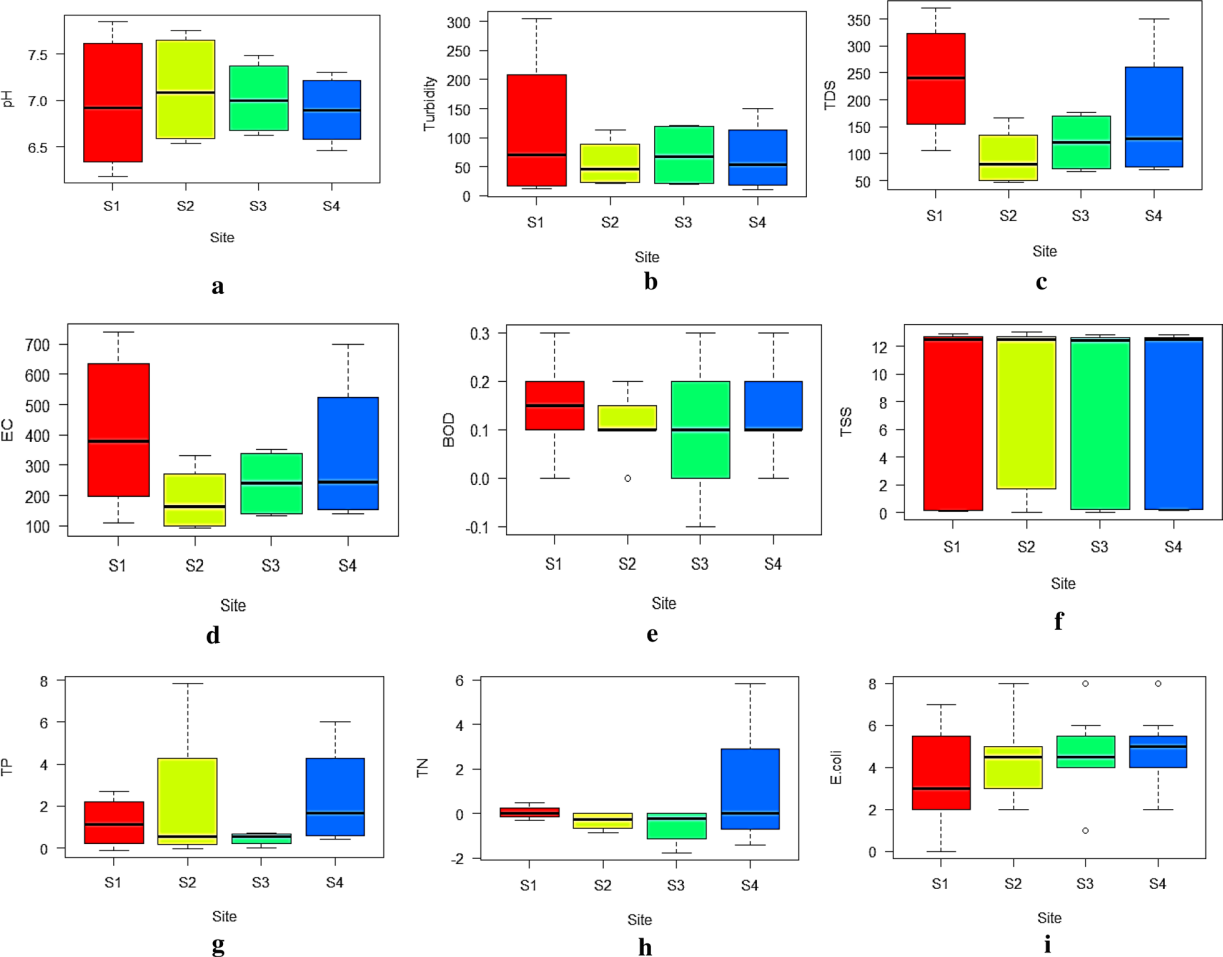
Box plots showing the variations in the mean values of **a** pH, **b** turbidity, **c** total dissolved solids, **d** electrical conductivity, **e** biochemical oxygen demand, **f** total suspended solids, **g** total phosphates, **h** Total nitrogen, and **i***Escherichia coli* count at the sampled sites along Namanve stream

Data normality test and non-parametric multivariate analysis results are presented in Table 1 and Additional file 1: Table S1. The results of analysis of water samples are shown in Fig. 2 (Additional file 2: Table S2).

**Table 1:** Results of Shapiro–Wilk normality test on the evaluated water parameters

Parameter	W	*p*-value
pH	0.91031	1.38E−03
Turbidity (FTU)	0.76652	2.46E−07
Total dissolved solids (mg/L)	0.85725	3.41E–05
Electrical conductivity (µS/cm)	0.82019	3.84E–06
Total phosphates (mg/L)	0.68501	7.29E–08
Total nitrogen (mg/L)	0.38729	4.14E–10
Total suspended solids (mg/L)	0.63872	1.28E–09
Biochemical oxygen demand (mg/L)	0.74279	5.29E–07
*Escherichia coli* count (CFU/100 mL)	0.9631	1.35E–01

Data with *p*-value less than or equal to *0.05* are not normal. All *p*-values rounded to 2 decimal places

### Discussion

Nearly all the water quality parameters were within WHO permissible limits [19]. The pH of the samples was between 6.2 at S1 to 7.9 at S2 (Fig. 2a). These values are comparable to 6.0 ± 0.10 to 6.87 ± 0.60 reported in Nakawa-Ntinda industrial area [2, 7]. Phiri et al. [20] and Wanasolo et al. [7] hinted that such slightly lower pH values in effluents from beverage factories as in the current study are due to the nature of the raw materials such enzymes, lactic acid, benzoic acid and yeasts that are commonly used in such industries. It should be noted that even within the acceptable pH range, slightly high pH causes water to have a slippery feel whereas slightly low pH may cause water to have a bitter or metallic taste [10].

Turbidity in this study ranged from 10.70 FTU at S1 in the dry period to 305.00 FTU in the wet period at S1. All the turbidity values recorded were more than 5 FTU recommended by WHO for water to be used for domestic purposes. In water, turbidity more than 5 TFU indicates the presence of high bacteria levels, pathogens or particles that can shelter harmful organisms from disinfection processes and this is supported by the high counts of *E. coli* recorded [10]. Muwanga and Barifaijo [5], and Walakira and Okot-Okumu [2] recorded very high values of turbidity in effluents from some food industries in Uganda than in this study and they speculated that these could be due to decomposing organic matter in the effluents.

Total dissolved solids recorded ranged from 47.02 mg/L at S2 during the dry periods to 370.70 mg/L at S1 during the rainy period. The high TDS recorded for S1 could be because this sample had majorly effluent being discharged into the stream. These values are within the acceptable values of 1000 mg/L and 1200 mg/L for drinking water and effluent discharge standards set by WHO [19], National Environmental Management Authority (NEMA) [21] and the Ugandan Ministry of Water and Environment [22] respectively. High TDS affects the aesthetic quality of water, interferes with washing operations and can be corrosive to plumbing fixtures.

Electrical conductivity on the other hand ranged from 92.5 to 740 μS/cm. Samples from S1 and S4 had conductivity of 740 µS/cm and 400 µS/cm which were above the recommended limit of 400 μS/cm by WHO [19]. The high mean conductivities at site S1 and S2 could be due to high levels of mineral ions released in the effluent [7]. These results clearly indicate that samples at S1 and S2 were considerably ionized and had the highest concentration of ions due to excess dissolved solids. This could be due to mobilization of conducting ions during the decay processes of organic materials in the stream and thermal mobilization of ions as the water temperature increased [23]. The values were however lower than those recorded in some streams of Nakawa-Ntinda industrial area [2, 7]. Similar trends in conductivity were reported in Kinawataka stream, its tributaries [7] and the inner Murchison bay of Lake Victoria [24].

Figure 2e shows the trend of biochemical oxygen demand (BOD) along the sampled sites of Namanve stream. The highest BOD was 0.006 mg/L recorded at S3 during the dry season. All the BOD values were much lower than the normal background level of 2–3 mg/L and BODs reported in other industrial complexes by previous investigations [2, 5]. A previous study [6] established that pollution loading (kg/day) from industries due to BOD accounted for 13% of the total estimated pollution loading of Lake Victoria. The lower levels of BOD recorded could be due to dilution effect and natural purification mechanisms such as respiratory breakdown, biological and physical uptake in the aquatic ecosystem [2]. The highest mean value at S3 could be due to the organic content released in the effluent while at S4, this could have been due to agricultural fertilizers and organic matter washed into the stream by run-offs [25]. This could as well be a result of accumulation of organic matter released in the effluent into the stream. The BOD at S1 could be corresponding to the release of organic matter in the effluents.

Suspended solids are defined as the concentration (mg/L) of inorganic and organic matter, which is held in the water column of a stream, river, lake or reservoir by turbulence [26]. The maximum and minimum values for TSS were 0.04 mg/L at S2 during dry conditions and 13.04 mg/L during rainy season respectively. These values were much lower than 100 mg/L recommended for drinking water. High levels of TSS in waterbodies can have deleterious impacts on the physical, chemical and biological properties of a waterbody [26]. However, the magnitude of the effect depends on the concentration, duration of exposure, chemical composition and particle size distribution of the solids, but also varies between organisms and between environments [26].

Total phosphate levels at all the sites were below the threshold levels according to the National standards and WHO guidelines with the highest concentration of 7.85 mg/L at S2. There were marked variations in the mean concentrations at the different sites. Application of phosphate fertilizers in the nearby gardens could have resulted in the concentration at S2. The lowest concentrations at S3 and S4 could have been due to the self-replenishing function of water in the stream, uptake by the vegetation and distance from the pollution site as suggested by previous authors [10, 27].

Total nitrogen levels in the stream ranged from 0.001 mg/L at S3 to 5.828 mg/L at S4. Total nitrogen was detected at S1 and S4 while S2 and S3 had undetectable TN concentrations (Fig. 2h). The mean values at S1 and S4 were observed to be far below the recommended limits, meaning TN has insignificant effect on water quality of Namanve stream. The concentration reported at S4 was higher than that at S1 and could be attributed to contamination from other sources such as channels connected to the stream and fertilizer from run off from the nearby gardens. Traces of TN at S1 could have been released in the effluent from the industries. Undetectable TN concentrations at S2 and S3 could be a result of self-cleansing mechanism of water in the stream and uptake by microbes along the stream. However, increase to excessive levels of nitrogen would be associated with many large-scale environmental concerns, including eutrophication of surface waters, algae blooms, hypoxia, acid rain, nitrogen saturation in forests and global warming.

In this study, *E. coli* counts were determined to check the microbiological safety of Namanve stream water. At all sites, *E. coli* counts were above the acceptable limit. The high levels of *E. coli* could be attributed to the run off that washes faecal matter from the nearby settlement area into the stream. Namanve stream is situated near Bweyogerere town which is densely populated and is associated with poor sanitation including open defecation. Th *E. coli* counts recorded could have also been a result of accumulation of faecal matter released in the effluent from the surrounding industries [4]. The vegetation along the stream has been cleared thus there is nothing to enhance the biological function of purifying the effluent discharged and water along the stream. The high counts of *E. coli* may also be originating from other non-point sources of contamination.

## Limitations

In this study, (i) some water quality parameters were not determined (ii) samples were taken within one-month span and therefore the levels of contamination reported might not fully reflect the contamination of this stream. (iii) we did not determine the heavy metal content of this stream, yet locals use water from it for washing automobiles and growing sugarcane. Thus, there might be potential risk of deleterious health effects associated with heavy metals stemming from use of water and consumption of sugar cane from this stream.

## Supplementary information

**Additional file 1: Table S1.** Results for non-parametric multivariate analysis of variance (MANOVA) of investigated water parameters.

**Additional file 2: Table S2**. Raw data for investigated quality parameters of water from Namanve stream, Kampala Industrial and Business Park, Uganda.

## Data Availability

The datasets supporting the conclusions of this study are included within the article (and its additional files).

## Abbreviations

BOD: Biochemical oxygen demand
EC: Electrical conductivity
*E. coli*: *Escherichia coli*
KIBP: Kampala Industrial and Business Park
TDS: Total dissolved solids
TN: Total nitrogen
TSS: Total suspended solids
WHO: World Health Organization
