# Supplementary material for: Effects of industrial effluents on the quality of water in Namanve stream, Kampala Industrial and Business Park, Uganda
Source: BMC Res Notes. 2020 Apr 16;13:220. doi: 10.1186/s13104-020-05061-x (PMC7164339; doi:10.1186/s13104-020-05061-x)
Supplement: Supplementary file 1 — Additional file 1: Table S1. Results for non-parametric multivariate analysis of variance (MANOVA) of investigated water parameters. [file 13104_2020_5061_MOESM1_ESM.docx]

Table S1. Results for non-parametric multivariate analysis of variance (MANOVA) of the investigated water parameters

| Tests | Test statistic | df1 | df2 | *p*-value | Permutation test *p*-value |
| --- | --- | --- | --- | --- | --- |
| ANOVA test *p*-value | 4.478 | 12.252 | 48.6593 | 0.000 | 0.000 |
| McKeon approx. for the Lawley Hotelling test | 40.511 | 27.00 | 11.5888 | 0.000 | 0.002 |
| Muller approx. for the Bartlett-Nanda-Pillai test | 4.019 | 32.803 | 34.625 | 0.000 | 0.000 |
| Wilks Lambda (Ʌ) | 12.632 | 27.00 | 24.0064 | 0.000 | 0.000 |

Thus, there were significant variations (*p < 0.05)* in the water quality parameters at all the sites. Therefore, the hypothesis of equality between factor levels (sites S1, S2, S3, and S4) and the hypothesis of equality using response variables: pH, Turbidity, Total Dissolved Solids, Electrical Conductivity, Biochemical Oxygen Demand, Total Suspended Solids, Total Phosphates, Total Nitrogen and *Escherichia coli* were rejected.
